# Supplementary material for: Association of plasma metabolites and diagnostic imaging findings with hepatic lipidosis in bearded dragons (Pogona vitticeps) and effects of gemfibrozil therapy
Source: PLoS One. 2023 Feb 3;18(2):e0274060. doi: 10.1371/journal.pone.0274060 (PMC9897564; doi:10.1371/journal.pone.0274060)
Supplement: S1 Table — Each category is given a score from 0–4 based on the percentage of change in the histologic section of evaluated liver. Subsequently, a cumulative grade is used to determine the severity classification. Estimations using 400X magnification. (DOCX) [file pone.0274060.s001.docx]

SUPPLEMENTAL MATERIALS:

**S1 Table.** Histological grading system and severity classification for changes associated with diffuse and panlobular hepatic lipid accumulation in bearded dragons (*Pogona vitticeps).* Each category is given a score from 0-4 based on the percentage of change in the histologic section of evaluated liver. Subsequently, a cumulative grade is used to determine the severity classification. Estimations using 400X magnification.

| **Categories** | **Description** | **Grade** | **Comments** |
| --- | --- | --- | --- |
| *Percent of hepatocellular vacuolation* | 0-5% is grade 0, 1 is >5-25%, 2 is >25-50%, 3 is >50-75%, 4 is >75% | 0-4 | Based on the percent of lipid vacuoles (independent of size) present in the hepatocytes compared to the percent of cytoplasm in the evaluated liver section |
| Hepatocellular swelling | ≥ 30 nuclei is grade 0, 29-16 nuclei is 2, ≤ 15 is 4 | 0,2,4 | The average number of nuclei in of 10 field of views (0.2mm^2^) were calculated to determine if hepatocellular swelling and compression of sinusoids was present distorting the view of the nuclei |
| *Fibrosis* | (<10% is grade 0, 1 is 10-25%, 2 is >25-50%, 3 is >50-75%, 4 is >75% | 0-4 | Based on the percentage of fibrosis in the histologic section of liver, rather than the specific histological type or pattern |
| **Severity Classification** | **Cumulative Grade** | **Comments (majority of cases)** | |
| *No-lipid* | 0 | <5% hepatocellular vacuolation without hepatocellular swelling or fibrosis | |
| *Mild* | 1-4 | >5% hepatocellular vacuolation without hepatocellular swelling or associated fibrosis | |
| *Moderate* | 5-7 | ≥50% hepatocellular vacuolation, associated hepatocellular swelling and minimal fibrosis | |
| *Severe* | ≥ 8 | ≥75% hepatocellular vacuolation with severe hepatocellular swelling and variable fibrosis | |

Abbreviation: n/a, not applicable
