# Supplementary material for: Association of plasma metabolites and diagnostic imaging findings with hepatic lipidosis in bearded dragons (Pogona vitticeps) and effects of gemfibrozil therapy
Source: PLoS One. 2023 Feb 3;18(2):e0274060. doi: 10.1371/journal.pone.0274060 (PMC9897564; doi:10.1371/journal.pone.0274060)
Supplement: Attachment — Submitted filename: Rebuttal letter 1 05-2022.docx [file pone.0274060.s003.docx]

**­­Dear PLOS ONE reviewers and editors,**

**Thank you for reviewing our manuscript. The authors accepted all edits throughout the manuscript and added missing manufacturer information and clarified the anesthesia protocol.**

**Journal Requirements**

We noted in your submission details that a portion of your manuscript may have been presented or published elsewhere. 
(Results were published in a DVSc thesis at the University of Guelph:
<https://atrium.lib.uoguelph.ca/xmlui/handle/10214/26230>)

Please clarify whether this publication was peer-reviewed and formally published. If this work was previously peer-reviewed and published, in the cover letter please provide the reason that this work does not constitute dual publication and should be included in the current manuscript.

Response: This manuscript has not been peer-reviewed and formally published. It is included in a Doctor of Veterinary Science (DVSc) thesis that was successfully defended and is available online for review.

**Reviewer 1:**

Reviewer comment line 503 **“**small sample, low power. Risk of type II error?”

Response: Authors agree that the sample size was low in this pilot study and so acknowledged this in the discussion. Line 716 modified to say: “These results did trend in the right direction with the hepatic echogenicity increasing in comparison to the fat pads in the moderate-to-severe class and could be statistically significant with a larger sample size.”

Reviewer comment line 659 “would suggest adding these references here”

Response: Additional references were not visible on the PDF for authors to add.

**Reviewer 2:**

Authors demonstrated here that BHBA and succinate were very good candidates as biomarkers for abnormalities in hepatic lipid metabolism of bearded dragons and that gemfibrozil was good for the treatment of fatty liver in bearded dragons. These findings are very important and valuable for understanding bearded dragons' abnormality in lipid metabolism. However, several points in examination of gemfibrozil's effects should be confirmed before publish as the followings, although finding biomarkers is good. In addition, authors should modify this manuscript according to basic format as a manuscript submitted to PONE, because this manuscript was not described in a standard format.

Response: Authors changed the format of the supporting information file from docx to pdf, and added a conclusions section.

Majors:

1) Authors should show CT data of gemfibrozil-treated bearded dragons. The data would be useful for diagnosis and treatment of fatty liver.

Response: All data is available in the repository link in the results section of the manuscript. In addition, the CT HU data are presented in the plots and the text. If you meant that more CT images should be present, this is certainly something we could add but representative CT images were already displayed.

2) Serum parameters of gemfibrozil-treated bearded dragons should be shown. An important effect of gemfibrozil is to decrease serum TG levels. Therefore, data on serum parameters such as TG levels are essential for evaluating the effects of gemfibrozil. If possible, the time course of TG levels during gemfibrozil administration should be indicated.

Response: All data is available in the repository link in the results section of the manuscript. Triglyceride levels were only measured once prior to gemfibrozil therapy and once after completion of therapy. Figure 9 shows box plots of these baseline and post treatment levels in the treatment and control group.

Minors:

3) The "Materials and Methods" section should be rearranged. In the "Results" section, animal information should not be described and only explanation of data should be described. The animal information should be moved to the "Materials and Methods." In the "Materials and Methods", "Inclusion criteria, animals, exclusion criteria" is too long and broken into small items including the animal information described in the "Results" now.

Response: The “Animals” section was removed from the results and incorporated into the materials and methods under “Coelioscopic guided liver biopsy and histology” as it describes the weight distributions of the different histological classes. “Inclusion criteria, animals, exclusion criteria” was shortened by removing sections on bearded dragon initial admission and parasite screening and cricket gut-loading.

We would like to keep some of the information on husbandry as it is often asked by reviewers or needed by readers to ensure biochemistry changes and other physiological parameters have not been confounded by an inappropriate husbandry (mainly environmental parameters) and diet. This information could also be helpful to researchers interested in keeping bearded dragons as laboratory animals.

4) Figure legends should be revised. Normally, figure legends should contain information on the display of data. The figure legends in this manuscript is too little information and difficult to understand. It is necessary to describe in Figure legends what kind of values, what kind of graphs, how to do statistical analysis, etc. Please check other articles on PONE carefully.

**Response: Authors modified figure legends to describe the kind of values, kind of graphs, and statistical analysis. Please see modifications made to the manuscript.**
